# Different selective pressures lead to different genomic outcomes as newly-formed hybrid yeasts evolve

**DOI:** 10.1186/1471-2148-12-46

**Published:** 2012-04-02

**Authors:** Jeff S Piotrowski, Saisubramanian Nagarajan, Evgueny Kroll, Alison Stanbery, Kami E Chiotti, Arthur L Kruckeberg, Barbara Dunn, Gavin Sherlock, Frank Rosenzweig

**Affiliations:** 1 Chemical Genomics Research Group, RIKEN Advance Science Institute, Wako, Wako, Japan; 2 School of Chemical and Biotechnology, SASTRA University, Tirumalaisamudram Thanjavur- 613401, Tamil Nadu, India; 3 Division of Biological Sciences, The University of Montana, Missoula MT 59812, USA; 4 DuPont Corporation, Wilmington DE 19880, USA; 5 Department of Genetics, Stanford University School of Medicine, Stanford, CA 94305-5120, USA

## Abstract

**Background:**

Interspecific hybridization occurs in every eukaryotic kingdom. While hybrid progeny are frequently at a selective disadvantage, in some instances their increased genome size and complexity may result in greater stress resistance than their ancestors, which can be adaptively advantageous at the edges of their ancestors' ranges. While this phenomenon has been repeatedly documented in the field, the response of hybrid populations to long-term selection has not often been explored in the lab. To fill this knowledge gap we crossed the two most distantly related members of the *Saccharomyces sensu stricto *group, *S. cerevisiae *and *S. uvarum*, and established a mixed population of homoploid and aneuploid hybrids to study how different types of selection impact hybrid genome structure.

**Results:**

As temperature was raised incrementally from 31°C to 46.5°C over 500 generations of continuous culture, selection favored loss of the *S. uvarum *genome, although the kinetics of genome loss differed among independent replicates. Temperature-selected isolates exhibited greater inherent and induced thermal tolerance than parental species and founding hybrids, and also exhibited ethanol resistance. In contrast, as exogenous ethanol was increased from 0% to 14% over 500 generations of continuous culture, selection favored euploid *S*. *cerevisiae *x *S. uvarum *hybrids. Ethanol-selected isolates were more ethanol tolerant than *S. uvarum *and one of the founding hybrids, but did not exhibit resistance to temperature stress. Relative to parental and founding hybrids, temperature-selected strains showed heritable differences in cell wall structure in the forms of increased resistance to zymolyase digestion and Micafungin, which targets cell wall biosynthesis.

**Conclusions:**

This is the first study to show experimentally that the genomic fate of newly-formed interspecific hybrids depends on the type of selection they encounter during the course of evolution, underscoring the importance of the ecological theatre in determining the outcome of the evolutionary play.

## Background

Interspecific hybridization occurs in every eukaryotic kingdom and can lead to reticulated rather than branching phylogenies [1,2]. Hybrid progeny are often at a strong selective disadvantage (e.g., they may be sterile or have reduced viability). However, in some instances the increased genome size and complexity of interspecific hybrids may result in greater fecundity and/or adaptive flexibility than either ancestral species [3], particularly at the edges of the ancestral species' range, where they are more likely to encounter stress [4]. This phenomenon is amply documented in the agricultural literature as well as in field-based evolutionary studies [1,2,5-10]. Laboratory studies of interspecific hybridization have largely been confined to the *Drosophila *species complex, where foundational studies have shaped our understanding of the genetic basis for pre-zygotic and post-zygotic reproductive isolation [11-13]. Long-term experimental studies aimed at discerning the evolutionary trajectories open to newly-formed hybrids under different types of selection are lacking, a knowledge gap due in part to the scarcity of hybrid eukaryotes that have the short generation time and ease of preservation needed to undertake experiments lasting hundreds of generations.

Experimental evolution studies using microbes have enlarged our understanding of the tempo of adaptive change [14-16], the shape of the adaptive landscape [17], and the manner in which genotypes navigate that landscape [18]. Increasingly, the budding yeast *Saccharomyces cerevisiae *has become a favored model for such studies because of its genetic tractability, arsenal of post-genomic tools, and homology of many of its genes to those in higher eukaryotes. Yeast's short generation time, simple, heterogonic life cycle and ease of preservation ideally suit it for studying evolution in the laboratory [16,19-21], where it has yielded insights into the physiology of adaptive traits [22] and how genome structure evolves under selection [23,24].

The 7 members of the *Saccharomyces sensu stricto *group, though closely related, have long been recognized as biological species by virtue of their post-zygotic reproductive isolation [25,26]. While *bona fide *representatives of each species are easily recovered from nature [27], retrospective comparative genomics studies [28,29] suggest that interspecific hybridization has occurred repeatedly during the group's evolutionary history. Indeed, the lager yeast *S. pastorianus*, which likely arose ~500-600 years ago [30], is a natural hybrid of two species, *S. cerevisiae *and the newly discovered *S. eubayanus *[26]. *Saccharomyces *hybrids have most often been studied with the aim of developing industrially useful traits [31,32], typically by focusing on the physiology of single clones. However, Grieg et al. (2002) showed that the *sensu stricto *group could also be used to investigate hybrid speciation in the lab [33]. Currently lacking, however, are prospective studies of interspecific hybrid evolution under different types of selection, using either single clones or, more realistically, populations of hybrid clones, such as one might expect to arise in natural hybrid zones.

The two most distantly-related members of the *Saccharomyces sensu stricto *group, *S*. *cerevisiae *and *S. uvarum *(formerly *S. bayanus var. uvarum *[26]), are largely syntenic, exhibit 80% and 62% nucleotide identity in coding regions and intergenic regions, respectively, and are thought to have diverged ~20 million years ago [34]. The two species differ in their stress tolerances, with *S. cerevisiae *being much more thermal tolerant [35-37] and slightly more ethanol tolerant [38]; *S. cerevisiae *x *S. uvarum *hybrids can exhibit greater ethanol tolerance than either parental species [39]. These genetic and phenotypic differences, coupled with the availability of genomic resources for both species, make *S. cerevisiae *x *S*. *uvarum *hybrids an attractive system in which to investigate how newly-formed hybrid genomes evolve under different types of stress. We therefore sporulated a *S. cerevisiae *x *S*. *uvarum *hybrid and mass-mated the progeny to create a pool of *S. cerevisiae *x *S. uvarum *homoploid and aneuploid hybrids that was used to found six replicate populations. Because fungi in nature are chronically nitrogen limited [40,41], experimental populations were evolved in a glucose-sufficient, nitrogen-limited 'common garden;' three were subjected to incremental increases in temperature, and three were subjected to incremental increases in ambient ethanol. Because the two ancestral species and their F1 hybrid exhibit differential sensitivity to temperature and ethanol, and because stress has been shown to increase mitiotic recombination, including chromosome missegregation [42], we hypothesized that the two selective pressures would lead to different genomic outcomes. As prior experiments in *S*. *cerevisiae *had shown that thermal tolerance confers cross-protection against other types of stress [37,43], we further hypothesized that hybrids evolving under temperature selection would not only become more thermal tolerant than their ancestors but also exhibit greater ethanol tolerance, and *vice versa*.

We tested these hypotheses using an integrated approach that combined physiological assays with analysis of genome structure by Clamped Homogeneous Electric Field (CHEF) electrophoresis and array-Comparative Genomic Hybridization (a-CGH). Consistent with our primary hypothesis, temperature selection resulted in loss of the *S. uvarum *genome from interspecific hybrids, while ethanol selection resulted in yeast that retained essentially both *S*. *cerevisiae *and *S. uvarum *genomes. Consistent with our secondary hypothesis, cross-protection to ethanol was evident in the temperature selected isolates; however, cross-protection to thermal stress was not observed among ethanol-selected clones. Thus, as hybrid populations evolve under different selection pressures their fate may depend not only on the outcome of competition between individual variants, but also on the outcome of competition between the ancestral genomes themselves.

## Results and Discussion

### Creation of the founder hybrid population and experimental design

**Figure 1:**
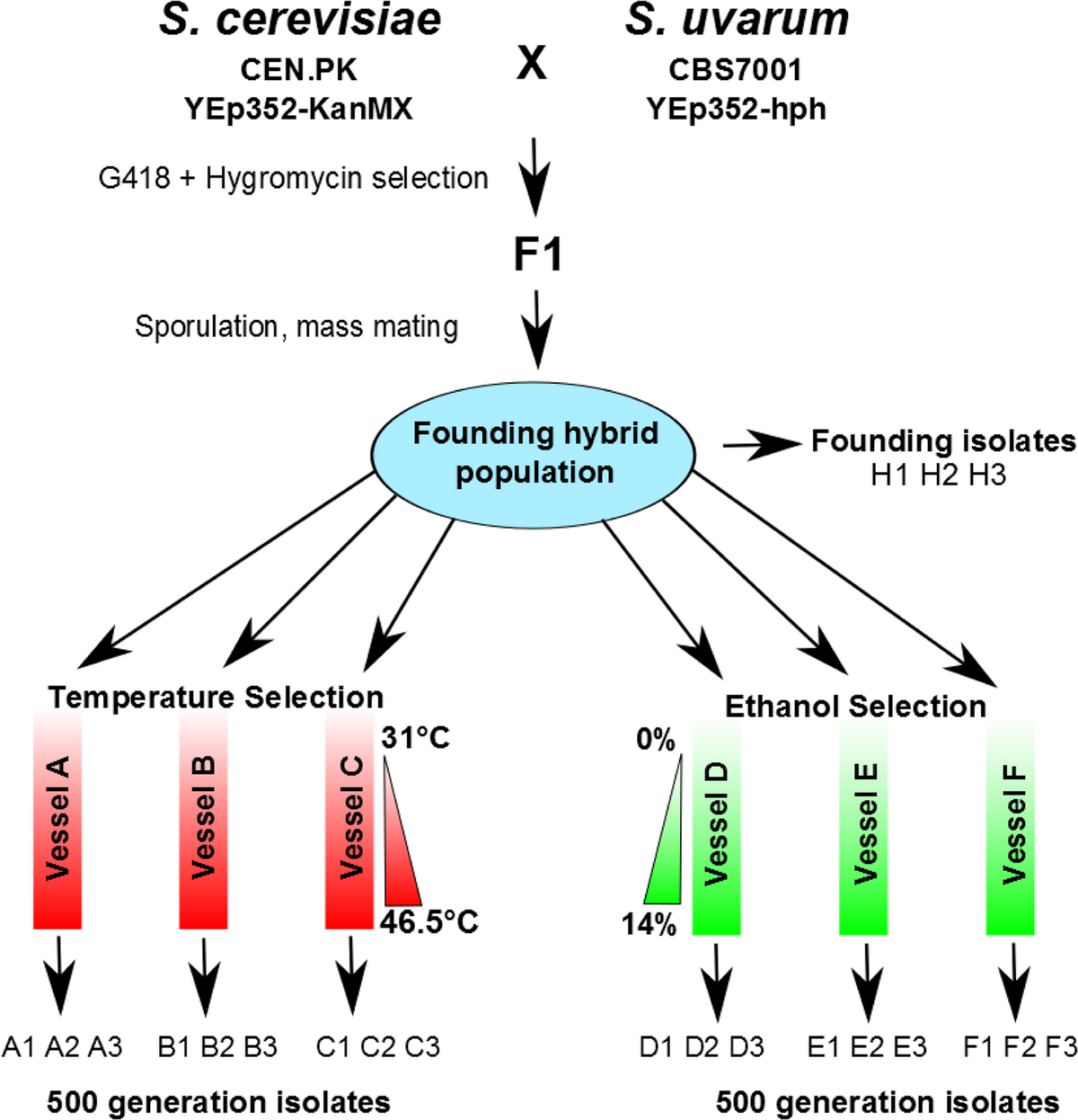
**Experimental design: Response of a genetically heterogeneous hybrid population to temperature or ethanol selection**. *S. cerevisiae *(CEN.PK) and *S. uvarum *(CBS7001) were transformed with plasmids conferring G418 and hygromycin resistance, respectively. The two species were mated, then placed under dual antibiotic selection to screen for a viable F1 interspecific hybrid. This F1 was sporulated and the spores allowed to diploidize by mass mating. The resulting genetically mixed hybrid population was used to found 3 experimental populations for temperature selection and 3 experimental populations for ethanol selection. After 500 generations of incremental increases in either temperature or ethanol, three clones were isolated from each experimental vessel and their stress tolerance compared to that of the two ancestral species, an F1 interspecific *S. cerevisiae*/*S. uvarum *hybrid, and three isolates from the founding hybrid population.

An overview of the experimental design is presented in Figure 1. A pool of approximately 10,000 interspecific hybrid clones was created using two prototrophic parental strains: CEN.PK (*Saccharomyces cerevisiae*) [44] and CBS7001 (*S. uvarum*) [30]. We crossed haploid derivatives of the parental strains using double antibiotic selection and verified by pulsed-field gel and by a-CGH that the resulting F1 hybrid contained a complete genome from each parent (data not shown). The hybrid population was then obtained by sporulating the F1 hybrid and removing vegetative F1 hybrid cells as described in Methods and in [33]. F1 hybrids sporulate at low efficiency, thus sporulation was done *en masse*. Because spore germination occurred in contact with other spores (see Methods), it is probable that most of the viable and mating-proficient gametes mated, resulting in a genetically mixed hybrid population that was largely diploid. Nevertheless, we cannot rule out the possibility that this population may have contained a low proportion of unmated F2 gametes. Additionally, despite our efforts to obtain a pure F2 population, some F1 cells did survive the procedure.

Hybrid cells with complete or almost-complete chromosomal complements tended to grow faster than those cells that were multiply aneuploid (data not shown). Consequently, our starting inoculum was a complex mixture of F1 and F2 hybrids having different levels of aneuploidy and specific growth rates. A mixture of F1, haploid and aneuploid F2 hybrids is an evolutionarily relevant starting point for our experiments, inasmuch as it mimics the genetic complexity one might expect to see within a hybrid zone [45]. We refer to this pool as the *founder hybrid population*. For purposes of comparison with clones subsequently isolated after many generations of selective growth, three isolates were randomly chosen from the founder hybrid population and denoted H_1_, H_2_, and H_3_. All strains used in this study are listed in Additional file 1: Table S1.

### Steady-state population size declines as evolving hybrid populations respond to increases in either temperature or ethanol

**Figure 2:**
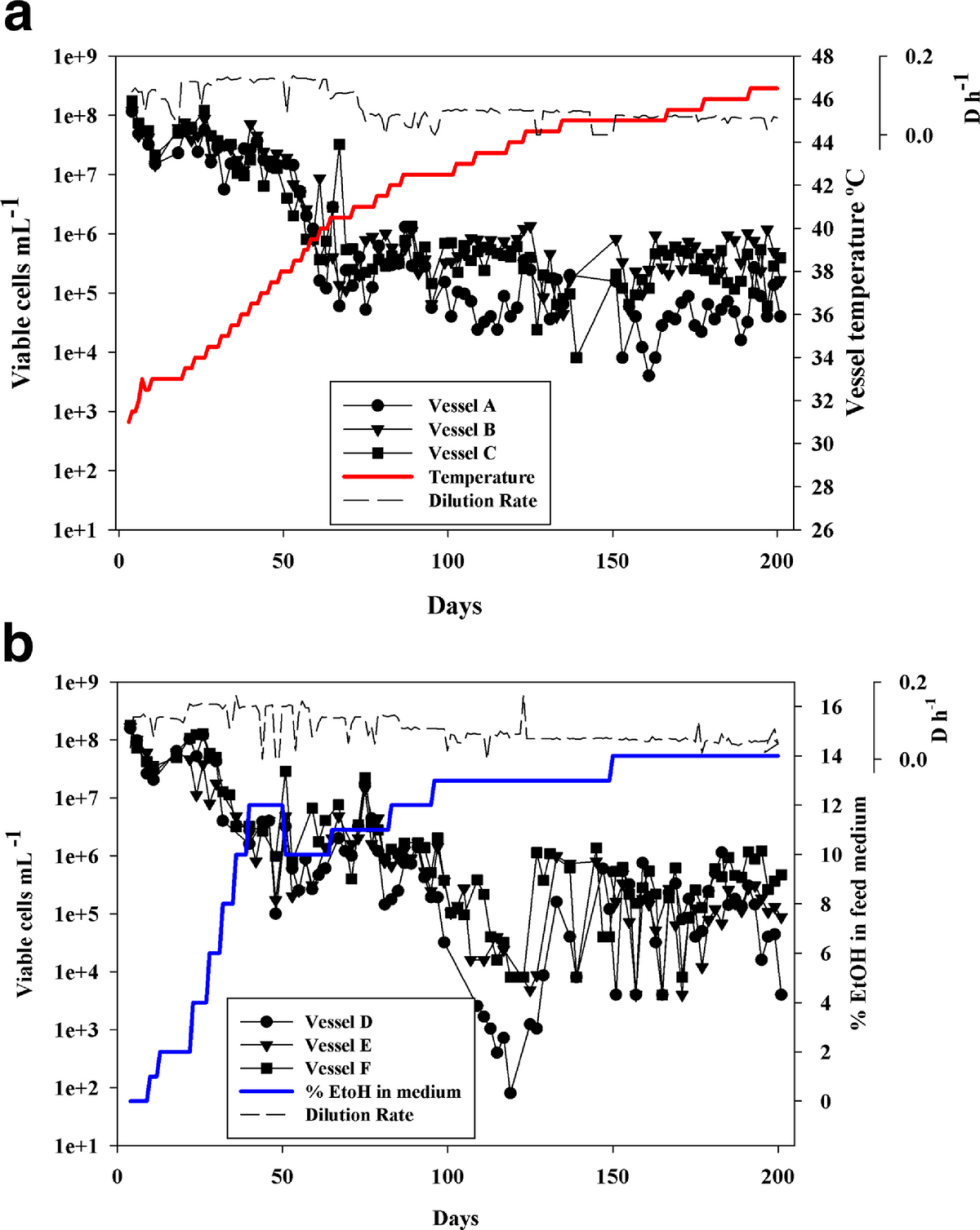
**Experimental evolution of *S. cerevisiae *X *S. uvarum *hybrids under increasing temperature (A) and ethanol (B)**. Six replicate populations, founded by the same genetically diverse hybrid pool (see Figure 1), were selected for 200 days (~500 generations) under glucose-sufficient, nitrogen-limiting conditions. Population size was estimated as viable cells mL^-1^. The dashed line represents dilution rate, D, in h^-1^; the solid red line represents vessel temperature **(A)**, the solid blue line represents ethanol content of the medium fed to evolving populations **(B) **(Mean ± S.E.).

In the wild, fungi including yeast are believed to live in a state of chronic nitrogen limitation [40], as do yeast in large-scale industrial fermentations [46,47]. To mimic these conditions we performed our selection experiments in a 'common garden' in which nitrogen in the form of ammonia was limiting. Cell density in evolving glucose-sufficient, NH_4_-limited populations declined as either temperature or ethanol was increased over the course of 500 generations (Figure 2). At the onset of experiments, cells cultured at 31°C attained steady state cell densities of 1.50 × 10^8 ^± 1.1 × 10^7^cells ml^-1 ^(N = 6, Mean ± S.E.). Following incremental temperature selection up to 46.5°C (Figure 2A) or ethanol amendment up to 14% (Figure 2B), steady state cell densities fell to 2.0 × 10^5 ^± 1.0 × 10^5 ^cells ml^-1 ^and 1.87 × 10^5 ^± 1.43 × 10^5 ^cells ml^-1 ^respectively, a decrease of 3 orders of magnitude. Indeed, at these low densities it was necessary to decrease chemostat dilution rate in order to prevent yeast populations from washing out of culture vessels, an indication of diminished growth rate under stress. We observed no dramatic between-vessel differences in population parameters, suggesting a similar response to ethanol and temperature selection in independent experiments founded with the same genetically heterogenous inoculum.

### Yeasts recovered from temperature selection are more thermotolerant than members of the founder population

**Figure 3:**
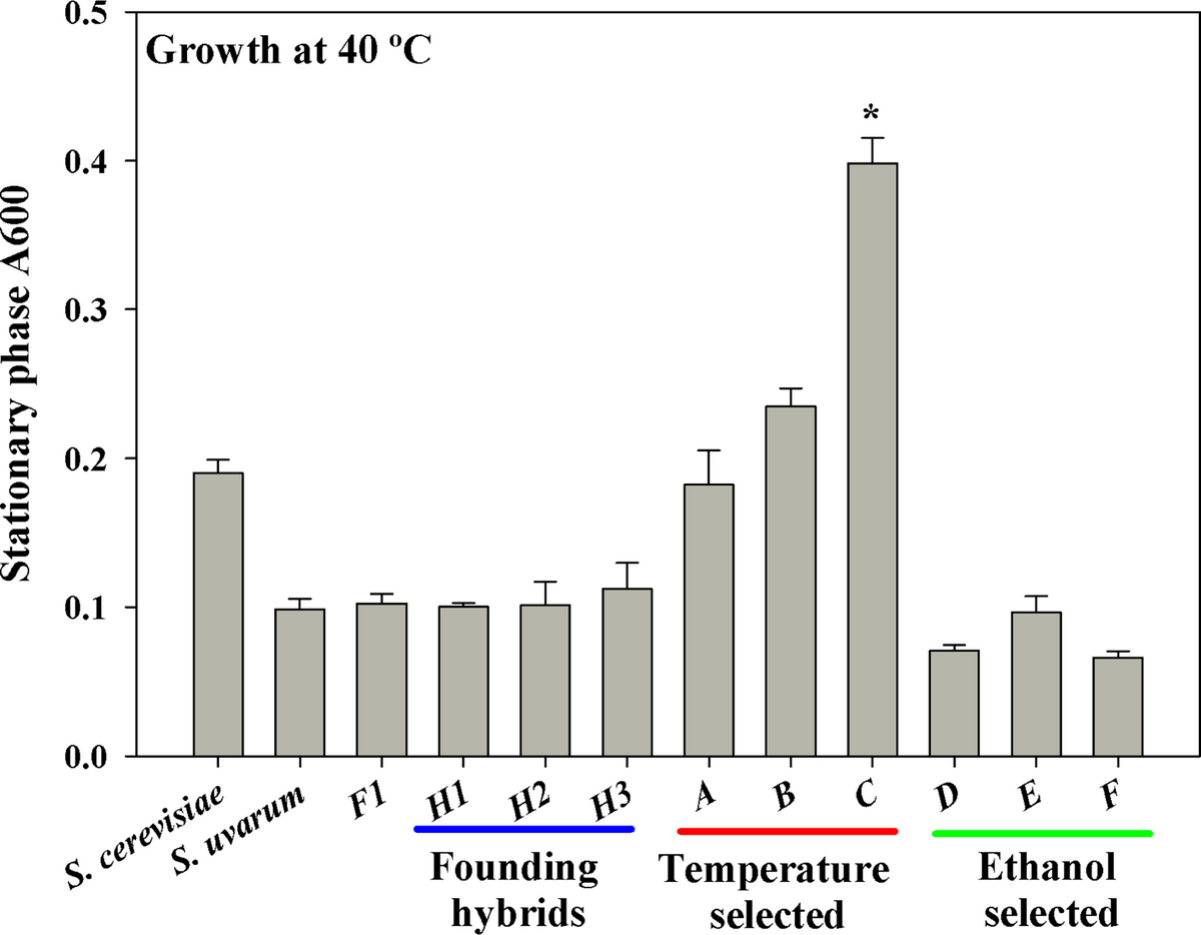
**Growth of evolved isolates at elevated temperature**. Culture density (A_600_) of parental, F1, founding and selected hybrid strains from each experimental population following 48 h growth in liquid, low-nitrogen, minimal medium at 40°C. Asterisk indicates significantly different growth, relative to all other isolates (*P *< 0.05, Mean ± S.E.).

To assess for heritable adaptations to thermal stress, we compared growth of 3 representatives of the founder hybrid population to that of 9 isolates selected at elevated temperature (3 single colony isolates from each experimental population, see Methods and Figure 1, Additional file 1: Table S1). When cultured at 40°C to stationary phase (48 h) in liquid low-nitrogen, minimal medium (Figure 3), temperature-selected isolates consistently showed greater thermal tolerance than *S. uvarum*, the F1 hybrid and members of the founder population. As a group, evolved isolates exhibited significantly greater yield than unevolved hybrids (*P *= 0.03, T-Test). Isolates from vessel C had significantly higher cell yield at 40°C than all other isolates including the *S. cerevisiae *parent (*P *< 0.05, ANOVA followed by Tukey's HSD).

**Figure 4:**
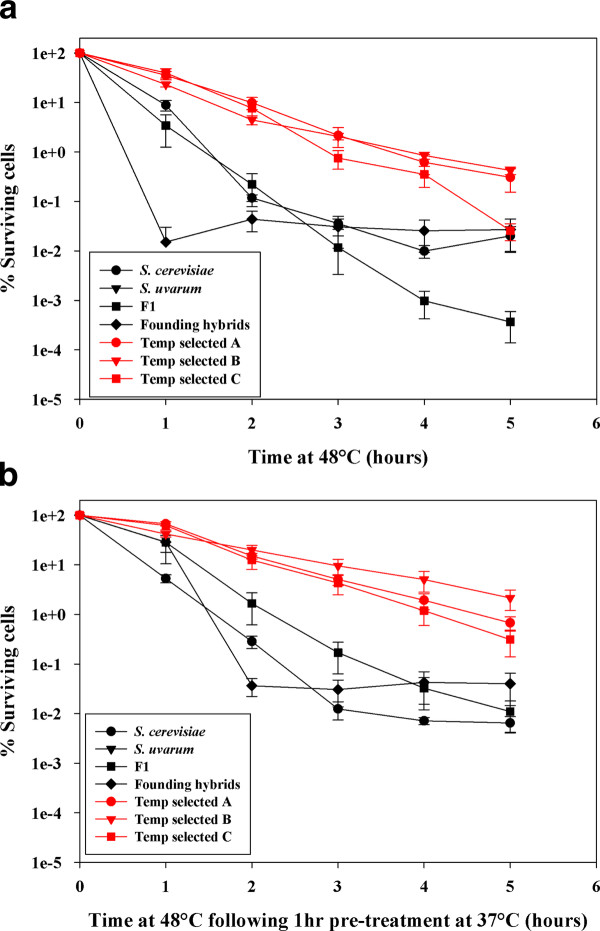
**Viability of parental species, F1 and founder interspecifc hybrids, and temperature-selected isolates in liquid culture following exposure to 48°C**. (A) Inherent thermal tolerance. A sample of each culture was diluted and plated on 2% YPD every hour for 5 h. No viable *S. uvarum *cells were detected at the 1 h time-point, thus *S. uvarum *data are not presented. After 2 h survivorship of the selected isolates was greater than all other isolates (*P *< 0.05) **(B) Induced thermal tolerance**. Following overnight culture at 25°C, cells were incubated for 1 h at 37°C prior to exposure to 48°C. Samples of each culture were then diluted and plated every hour for 5 h. No viable *S. uvarum *cells were detected at the 1 h time-point, thus *S. uvarum *data and are not presented. After 2 h, survivorship of the selected isolates was greater than the founding hybrids (*P *< 0.05). Red lines and filled symbols represent the temperature-selected isolates. Experiments were performed in triplicate (Mean ± S.E.).

Yeast acquire thermal tolerance following entry into stationary phase [48], as well as following exposure to low levels of heat or other types of stress [49,50]. Natural and laboratory variants can also exhibit strain-specific differences in thermal tolerance owing to different genetic backgrounds [35]. To test whether selection had resulted in yeast that were inherently more thermal tolerant than members of the founder population we evaluated survivorship of cultures exposed to 48°C for 5 h (Figure 4A). Following 1 and 2 h incubation at 48°C, all temperature selected isolates exhibited significantly greater survivorship than their ancestral strains (*P *< 0.05, Tukey's HSD). As no *S. uvarum *cells survived even 1 h at 48°C, they are not represented on this graph. We also wished to test the hypothesis that temperature-selected isolates would exhibit greater phenotypic plasticity under thermal stress. To do this, we induced thermal tolerance by first exposing cells to a sub-lethal elevated temperature (37°C) for 1 h prior to exposure to a 48°C heat shock (Figure 4B). Aside from *S*. *uvarum*, which again had no surviving cells, all other strains exhibited thermotolerance. Temperature-selected clones from populations A and C displayed higher survivorship than the *S. cerevisiae *parent and representative hybrids from the founding hybrid population at 2 h (*P *< 0.05, Tukey's HSD); clones from vessel B had significantly greater survivorship than founding hybrids (*P *< 0.05, Tukey's HSD). Thus, selection on a population of interspecific hybrids resulted in yeast that not only sustain growth at higher temperature than members of the founder population, but also are intrinsically more heat-shock resistant and able to acquire greater heat-shock resistance physiologically by induction.

### Yeasts recovered from ethanol selection do not appear to be more ethanol tolerant than members of the founder population

The manner and extent to which interspecific hybrid populations responded to ethanol selection was less clear-cut. When cultured until stationary phase (48 h) in liquid, low-nitrogen minimal medium amended with 8% ethanol, culture densities among ethanol-selected isolates were statistically indistinguishable from those of *S. cerevisiae*, the F1 interspecific hybrid, founder population isolates H1 and H2, and the temperature-selected isolates (Additional file 2: Figure S1). Only the *S. uvarum *parent and founder isolate H3 proved to be ethanol sensitive by this assay (*P *< 0.05, Tukey's HSD). Furthermore, temperature selected isolates were no less ethanol tolerant than ethanol-selected isolates.

### Temperature and ethanol selection can lead to changes in cell wall integrity

**Figure 5:**
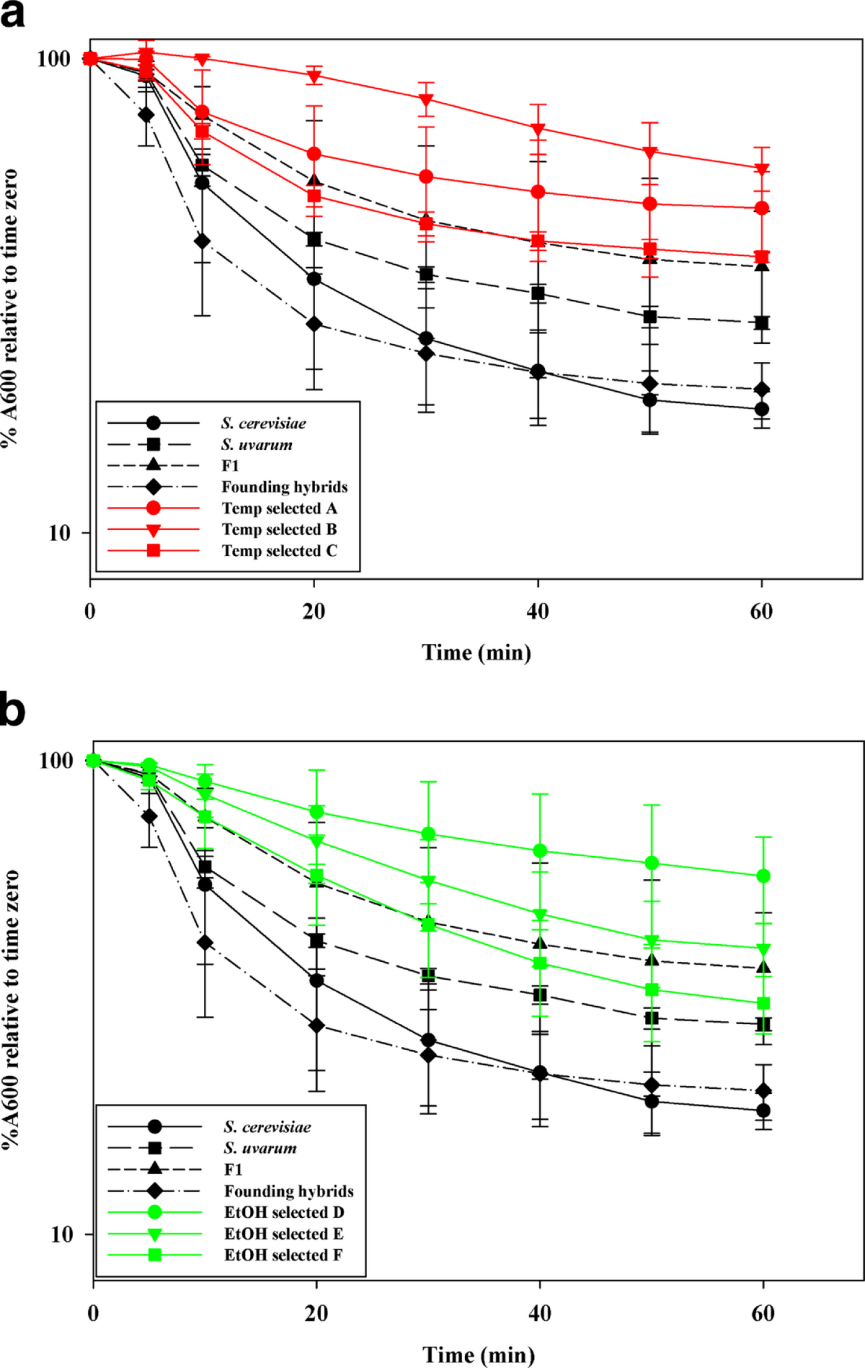
**Resistance of parental, founding hybrid, and (A) temperature-selected or (B) ethanol-selected hybrid yeast to zymolyase**. Cell wall dissolution was assayed by exposing yeast to zymolyase, placing them in spheroplast buffer at 37°C, then monitoring decrease in absorbance over time at λ = 600 nm, relative to T_0_. Each point is the mean (n = 3) of isolates from each selection vessel (1 from each vessel), 3 founding isolates, the F1, or parental isolates (Mean ± S.E.).

Heat-shock studies in *S. cerevisiae *have shown that yeast in prolonged stationary phase become resistant to heat and other stresses owing to progressive changes in cell wall structure that alter its mannoprotein content, increase the amount of chitin incorporated and augment the β-1,6 glucan fraction [51,52]. Phenotypically, such cell wall changes manifest as increased resistance to zymolyase digestion [51]. We therefore evaluated resistance of ancestral and evolved yeast to zymolyase digestion using the spheroplast assay described by [53]. Over the course of a 1 h incubation, the cell walls of temperature-selected isolates from vessels A, B, and C consistently exhibited higher average resistance to enzymatic dissolution than did either parental strain, the F1, or isolates from the founder hybrid population (Figure 5A). As a group, temperature-selected strains had significantly higher remaining absorbance percentage (48 ± 6%) after 1 h than founder population hybrids (20 ± 4%), (*P *< 0.05, T-Test).

We also observed that temperature-selected isolates were more resistant to Micafungin, a drug that targets β-1,3 glucan synthesis [54] (Additional file 3: Figure S2). Isolates from vessels A and C exhibited significantly greater resistance to this drug than *S. cerevisiae, S*. *uvarum*, the F1 and founding hybrids (*P *< 0.05, Tukey's HSD). This suggests that changes in cell wall composition or deposition contribute to the thermal tolerant phenotype observed in isolates from vessels A and C, whereas isolates from vessel B may have evolved a different resistance mechanism. This suggestion is supported by the fact that vessel B isolates, while not Micafungin-resistant, did exhibit resistance to zymolyase digestion.

Although interspecific hybrid populations responded weakly, if at all, to ethanol selection, we nevertheless assayed for heritable changes in cell wall integrity. Interestingly, similar to members of the temperature-selected populations, ethanol-selected isolates were more zymolyase resistance than parental species, the F1 hybrid and founder isolates (Figure 5B), though only isolates from vessel E showed significantly greater zymolyase resistance than founding hybrids after 1 h of treatment (*P *< 0.05, T-test). Strains isolated from ethanol-selected populations showed no difference in Micafungin sensitivity relative to the parental species, the *S. cerevisiae *x *S. uvarum *F1 hybrid, or isolates drawn from the founder population (Figure 5B).

### Temperature and ethanol selection lead to different genomic outcomes

**Figure 6:**
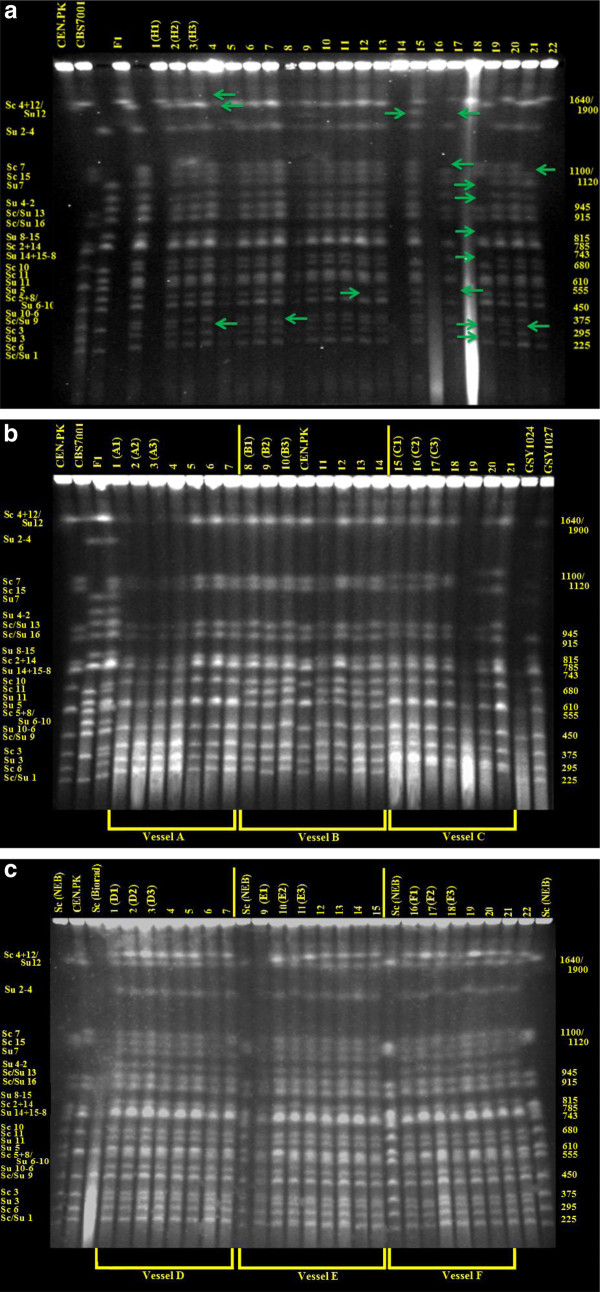
**(A) CHEF karyotypes of the founder population, 25°C**. At left are the karyotypes of parental strains, *S. cerevisiae *CEN.PK, *S. uvarum *CBS7001, and their F1 interspecific hybrid. In lanes 3-24 are a set of random clones isolated from the common interspecific hybrid pool used to found all replicate populations. Green arrows indicate instances of karyotypic diversity **(B) CHEF karyotypes after 500 generations of nitrogen limited, glucose sufficient culture with increasing temperature**. 7 random clones were isolated from each experimental population. **(C) CHEF karyotypes after 500 generations of nitrogen limited, glucose sufficient culture with increasing ethanol**. 7 random clones were isolated from each experimental population. Three different *S. cerevisiae *reference markers are included: the parent CEN.PK, one from New England Biosystems (Sc NEB), and from Biorad (Sc Biorad).

We initially assessed genome-wide changes in evolving hybrid populations by analyzing their electrokaryotypes using Contour-clamped Homogeneous Electric Field (CHEF) electrophoresis. CHEF analysis indicated that the founding hybrid population consisted of a mixture of F1 and F2 spore progeny (Figure 6A). Following 500 generations of temperature selection under nitrogen limitation (Figure 6B), populations in vessels A and B appeared to be karyotypically monomorphic, whereas the population in vessel C was polymorphic (e.g., Figure 6B, lane 20), albeit with a numerically dominant clone. Interestingly, the numerically dominant karyotype in each population (e.g., Figure 6B, lane 7) could be readily distinguished from the dominant clone in each of the other two populations (e.g., Figure 6B, lanes 8 and 17), indicating that while cell growth phenotypes might have been similar among vessels, genome content was not. In other words, different genomic architectures can solve the problem of growing at elevated temperature under nitrogen limitation. Further inspection of the karyotypes suggested that clones from populations in vessels A and C had lost virtually all the *S. uvarum *chromosomal complement of their genomes, keeping only the *S. cerevisiae *genome, while isolates from vessel B retained not only the entire *S. cerevisiae *genome, but also an additional chromosome (Figure 6B, lane 8). The dramatic large-scale genomic changes observed during temperature selection contrast sharply with ethanol selection, where after 500 generations, hybrids showed few rearrangements and no apparent loss of either parental genome (Figure 6C**)**.

### Temperature selection on the founding hybrid population favors loss of the *S*. *Uvarum *genome

**Figure 7:**
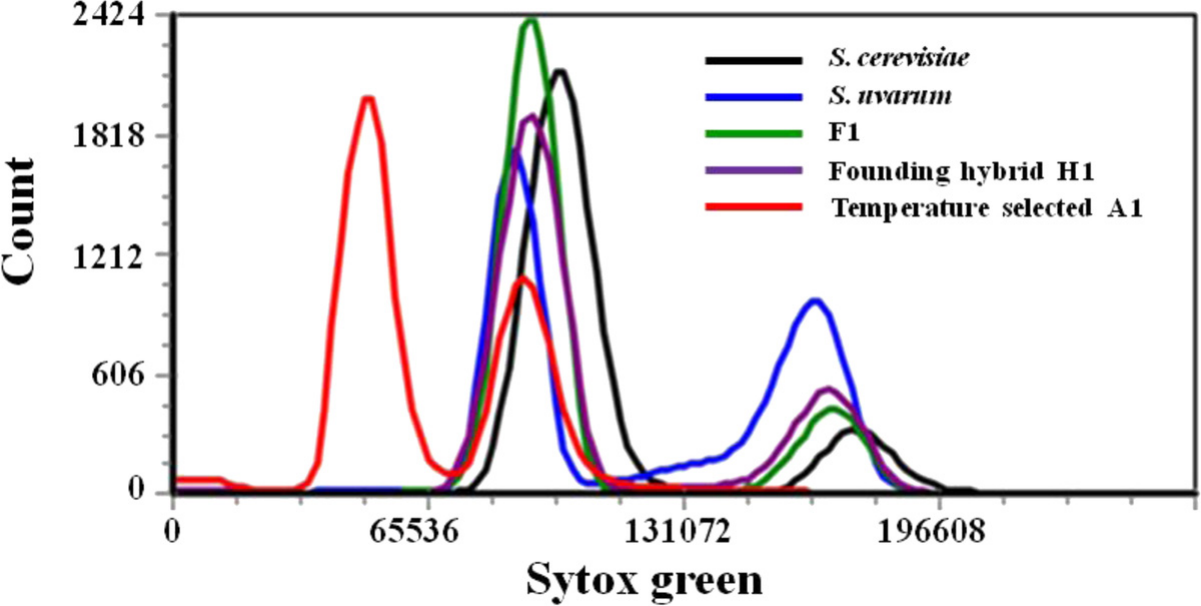
**Ploidy of temperature-selected isolates differs from that of parental species, the F1 interspecific hybrid, and a founder hybrid**. A representative isolate from Vessel A is presented. The transition from diploidy to haploidy in each temperature-selected population is presented in Figure S3. Cells were stained with SYTOX and sorted by flow cytometry.

Because changes in cell size and budding pattern (data not shown) suggested that temperature-adapted interspecific hybrids had become haploid, genome content was assessed by flow cytometry of SYTOX green-stained cells. As anticipated, the parental species, the F1 interspecific hybrid, and a representative from the founder hybrid population were all diploid, whereas temperature-selected clone A1 was haploid (Figure 7). To estimate the approximate number of generations required for haploid cells to become prevalent in experimental populations we performed flow cytometry on archived population samples (rather than on single clones). We found that although populations in vessels A, B and C were founded with the same inoculum, the transition from diploidy to haploidy occurred at a different time in each (Additional file 4: Figure S3). The last point at which population A was predominantly diploid was 101 generations (37.5°C), population B, 115 generations (37°C) and population C, 43 generations (33°C). Moreover, the number of additional generations subsequently required for haploid, or nearly haploid genomes, to become prevalent differed among replicate experiments. An additional 123 generations ensued before population A became mostly haploid, whereas this occurred in population B within 20 generations. And although haploids arose earliest in population C, 52 additional generations elapsed before the population became mostly haploid. Because the kinetics of transition between diploidy and haploidy differed among populations, fitness relationships between haploids and diploids co-evolving in each vessel likely came to differ, even though all were founded with the same inoculum and maintained under identical conditions.

We speculate that haploids arose when diploid hybrids sporulated under aerobic nitrogen limitation. Alternatively, a rare haploid, or nearly-haploid, F1 gamete may have been present in the initial founder hybrid population. If such a gamete contained only *S. cerevisiae *chromosomes and no *S. uvarum *chromosomes, it would have been HO- (and separated from potential opposite-mating-type spores by turbulence in the liquid medium), and could therefore have remained haploid. Whatever their origin, multiple temperature-selected clones exhibited chromosome rearrangements at 500 generations (Figure 6B). *S. cerevisiae*-like haploids likely acquired a competitive advantage at temperatures exceeding 35°C, a speculation supported by reports that the cardinal growth temperature of *S. cerevisiae *is significantly greater than *S. uvarum *[35,36] and (Figure 3). Given that temperature tolerance is a multi-locus, quantitative trait [55], haploidization resulting in nearly-complete loss of the *S. uvarum *genome complement provides the shortest path to thermal tolerance for *S. cerevisiae *X *S. uvarum *hybrids. Further, haploid *S. cerevisae *are known to exhibit greater induced thermotolerance than isogenic diploids [56], and haploids outcompete diploids at elevated temperature (37°C) when serially propagated in either YPD or low-nitrogen, minimal medium [57]. Consistent with these observations, after 48 h of growth at 40°C the haploid form of our *S. cerevisiae *parent attained higher cell density than the diploid (A_600 _= 0.28 ± 0.01 vs. 0.19 ± 0.01).

### Two species array comparative genomic hybridization supports the inference that genome evolution in interspecific hybrid populations is selection-specific

**Figure 8:**
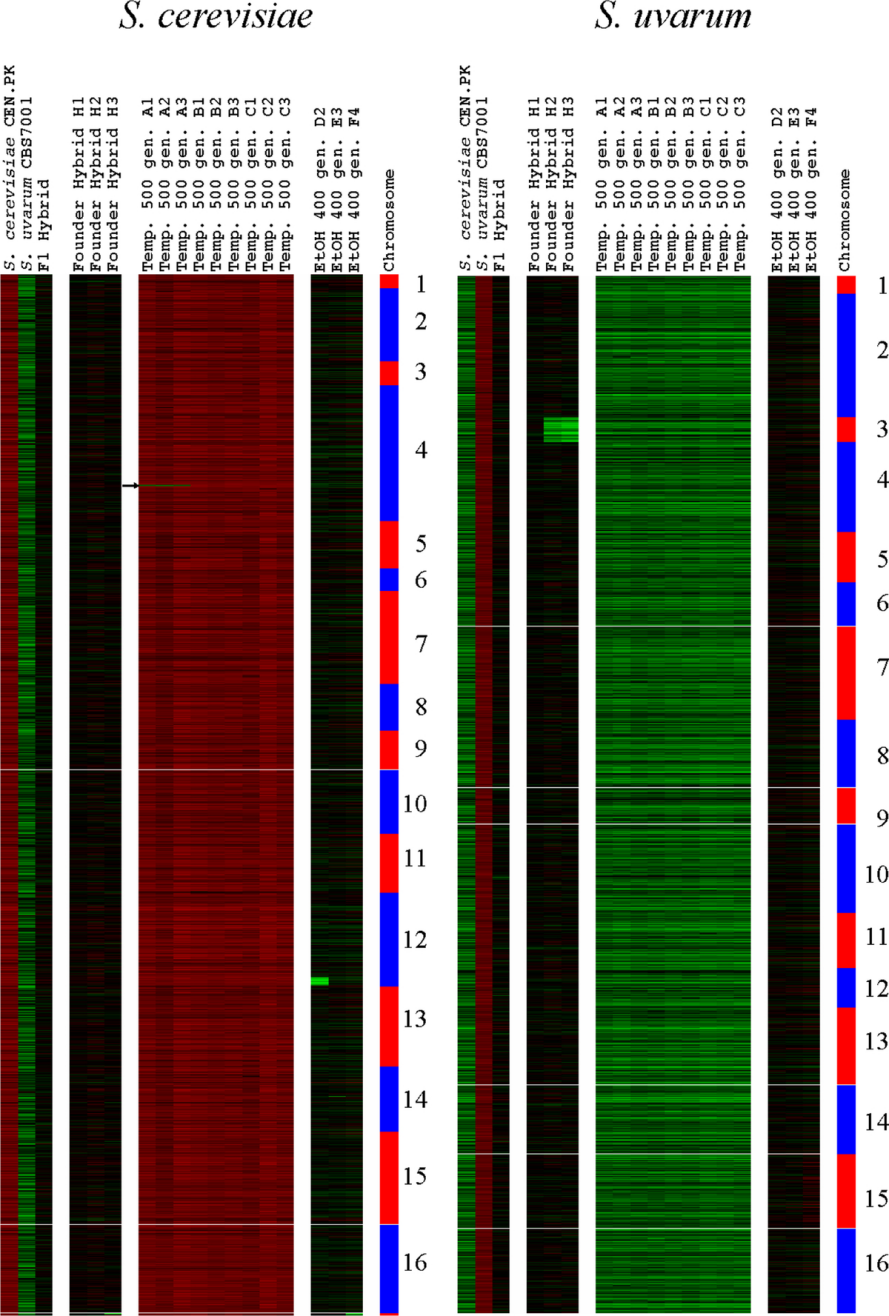
**Array-CGH data for parents, F1, representative founders, and temperature-and ethanol-selected clones**. Each column contains the a-CGH hybridization data for a given strain, while each row corresponds to a probe for a chromosomal location. Probes are ordered downward (for each parental genome separately as shown at top) from the left end of Chromosome I (top-most probe) to the right end of Chromosome XVI (bottom probe); note that probes for the *S. cerevisiae *mitochondrion are shown below its Chromosome XVI. Strains that show most of their hybridization intensities as a **red **color indicate the **presence **of most or all of the "red" parental species' genome, concomitant with the **absence **(**green**) of all or most of the other species' genome, whereas hybridization intensities appearing as black indicate a balanced complement of both parental species' genomes. The arrow indicated a deletion is located on Chromosome IV and corresponds to the ARS in the region between *HXT6 *and *HXT7*.

To further characterize genomic changes that may have occurred during evolution at elevated temperatures or elevated ethanol, we performed array-Comparative Genomic Hybridization (a-CGH) on the temperature- and ethanol-selected isolates as well as on parental strains and founding hybrids, using microarrays designed to uniquely detect hybridization to either the *S*. *cerevisiae *or the *S. uvarum *genomes, as described in [30]. Analysis of the parental strains indicates that our *S. cerevisiae *and *S. uvarum *isolates appear exactly as expected, i.e., they contain full genomes of their respective species with no chromosomal regions corresponding to the alternate species (Figure 8). The parental F1 hybrid contains, also as expected, a full set of chromosomes from both its *S. cerevisiae *and *S. uvarum *parents (Figure 8). Three isolates from the founder hybrid population were examined by a-CGH (See Additional file 1: Table S1). One (H1) appears to contain a complete two-species genomic complement, and is thus possibly an F1 hybrid (Figure 8), consistent with its CHEF electrokaryotype (Figure 6A, lane 1). Two other isolates from the founder hybrid population (H2 and H3) lack *S. uvarum *Chromosome III (Figure 7), as was also seen by CHEF. At the level of resolution afforded by a-CGH we see no evidence for recombination between parental genomes in (Figure 7). Indeed, without a separate study on recombination in *S. cerevisiae *x *S. uvarum *hybrids it would be impossible to distinguish between an F1 that lost *S. uvarum *Chromosome III and an F2 that retained the majority of both parental genomes. Nevertheless, even given the likelihood of suppressed recombination between homeologous chromosomes, the founding hybrid population was genomically diverse, owing to random segregation of these homeologous chromosomes when the F1 hybrid underwent meiosis.

Using a-CGH we examined the same 9 temperature-selected isolates described above (A1 through A3, B1 through B3, and C1 through C3, Figure 1). For all 9 clones, the genome complement appears to be comprised solely of that of the *S. cerevisiae *parent (Figure 8). The a-CGH results for the three vessel A clones thus closely agree with their CHEF gel results (Figure 6B); furthermore, a-CGH reveals that all three vessel A clones contain a small deletion in the *S. cerevisiae *genome; because its length is only ~1-3 kb it was undetectable on CHEF gels. This deletion is located on Chromosome IV and corresponds to the ARS in the region between *HXT6 *and *HXT7 *(Figure 8, indicated by an arrow). As the deletion appears identical among the three clones within the detection limits of a-CGH, it likely arose from the same member of the founder hybrid population within population A and does not represent multiple independent evolutionary events. Additional experiments will be required to ascertain both its prevalence in the vessel A population (i.e., whether it is "fixed"), as well as its possible adaptive value.

For the three 500-generation clones from population B and C, our a-CGH results do not intuitively agree with the CHEF results. CHEF gel karyotypes for all B clones appear to show the presence of an additional chromosome; although the band is similar (but not identical) to mobility of *S. uvarum *Chromosome XI, a-CGH results from all assayed 500-generation vessel B clones show the presence of only the parental *S. cerevisiae *genome (Figure 8) indicating that the band must derive from a copy-neutral event within the *S. cerevisiae *genome. Similarly, the CHEF electrokaryotypes of the clones from population C show increase in size of at least two of the smaller chromosomes, but again, only the parental *S*. *cerevisiae *genome is present (Figure 8), suggesting the occurrence of copy-neutral rearrangements.

We also performed a-CGH on 3 ethanol-selected isolates from each of Vessels D, E, and F (Figure 7). We investigated clones from the 400 generation time-point rather than at 500 generations, as we had observed greater karyotypic diversity in the 400 generation populations (Figure 6C vs. Additional file 5: Figure S4). An ethanol-selected clone from vessel D (Figure 7, EtOH 400 gen D2) appeared to be an essentially euploid F1 hybrid with a large deletion in the *S. cerevisiae *genome (but not deleted in the corresponding *S. uvarum *region): 100 kb of the right end of Chromosome XII, starting just proximal to (and including) the *TUS1 *gene and extending to the right telomere, is deleted in this isolate. Interestingly, Tus1 is involved in cell integrity signalling [58] and its deletion results in increased chitin deposition [59]; *ECM30*, which is also included in the deleted region, is a gene possibly involved in cell wall biosynthesis. This deletion may explain the increased zymolyase resistance observed in Vessel D isolates (see Figure 5B). The Vessel E isolate examined by a- CGH again appeared to be an essentially euploid F1 (Figure 8, EtOH 400 gen E3**)**, but exhibited a 15 kb deletion on its *S. cerevisiae *Chromosome XIV starting between *MEP2 *and *AAH1 *and extending to a tRNA gene just beyond *FYV6*. The deleted region contains genes involved in a variety of functions including nitrogen utilization (*AAH1*), recombination (*THO2 *and *FYV6*) and chromatin modifications (*EAF7 *and *FPR1*). The ethanol-selected isolate from Vessel F lost its *S. cerevisiae *mitochondrial genome, yet otherwise appeared as an intact euploid F1 hybrid (Figure 8, EtOH 400 gen F4**)**.

## Conclusions

This study is the first to report how populations of interspecific hybrid organisms evolving in the laboratory follow dramatically different evolutionary trajectories depending on the selective pressures applied. Indeed, to the best of our knowledge this is the first long-term study in experimental microbial evolution initiated with a heterogenous population of genetically diverse variants rather than with a homogenous population derived from a single clone. This collection of variants was used to found six replicate populations that evolved under glucose-sufficient, nitrogen-limiting conditions: three under temperature selection and three under ethanol selection.

In clones that arose under temperature selection, thermal tolerance was significantly greater than that of isolates in the founder population and of the parental F1 strain, a result likely due in part to segregational loss of the temperature-sensitive *S. uvarum *genome, resulting in *S*. *cerevisiae *haploids. This event occurred independently in all populations under temperature selection, although the haploid variants arose at different times and swept their respective populations over different time intervals. Interestingly, loss of heterozygosity under thermal and oxidative stress has recently been documented in *Candida albicans*, where increased stress appears to elevate rates of recombination, including chromosome missegration [42]. In diploid *S. cerevisiae*, haploidization has now been shown to provide an escape for persistent DNA rearrangement stress due to the presence of mutator alleles [60]. Thus, strong selection to augment thermal tolerance as well as to diminish DNA stress may help to explain the outcome of temperature selection on a population of newly-formed interspecific hybrids.

Heritable thermotolerance among these strains was evident in their ability to grow at temperatures restrictive to founding hybrid isolates on solid (data not shown) and in liquid media, as well as in their intrinsic and acquired resistance to heat shock. Temperature-selected strains were also ethanol resistant, most likely due to loss of the ethanol-sensitive *S*. *uvarum *genome [38]. Based on CHEF analysis, two of three temperature-selected populations came to be dominated by a unique karyotype, while one remained polymorphic, indicating multiple pathways to the evolution of thermotolerance. Evolution of multiple heat stress tolerance mechanisms was also manifest as differential, strain-specific resistance to zymolyase digestion as well as to Micafungin, a drug that targets cell wall biosynthesis. Because increased zymolyase resistance correlates with increased ethanol resistance [61,62], adaptive changes in cell wall integrity in temperature-selected clones may underlie "cross-protection" against ethanol stress.

Under temperature selection, adaptive changes in zymolyase and Micafungin resistance could be expected inasmuch as cell wall integrity has been shown to play a key role in thermal stress response [63]. Acquisition of thermotolerance in diauxic and stationary phase yeast has also been linked to accumulation of the disaccharide trehalose [64,65], however, we uncovered no evidence that trehalose hyper-accumulates in heat-shock resistant clones that arose under temperature selection (data not shown).

We uncovered little evidence for adaptive evolution of ethanol tolerance. Populations evolved under ethanol selection became dominated by euploid *S. cerevisiae *x *S. uvarum *hybrids. These hybrids showed improved growth on ethanol-amended media relative to some (e.g., founder H3), but not all founding hybrids, nor to the parental F1 strain. Evidence suggesting heritable changes in cell wall composition in these isolates was limited to higher (but not significantly higher) zymolyase resistance. We therefore conclude that a subset of euploid hybrids in the original founder population was at or near a fitness peak for ethanol tolerance [38], providing limited scope for selection.

It is important to bear in mind that temperature and ethanol tolerance assays were performed on only a few clones that together represent a small fraction of the genetic variation latent in our terminal populations. Other clones may exist that exhibit even greater thermal tolerance and, perhaps also greater ethanol tolerance. Indeed, in follow-up experiments we found that the temperature-selected clones we had randomly chosen for analysis grew poorly in chemostat monoculture at > 45°C, suggesting that other more highly tolerant variants exist in the terminal populations. Also, it may be that a slow ramp-up in temperature may be required even for thermotolerant clones to achieve their true performance maxima. We acknowledge the possibility that highly stress tolerant variants may have been present in the hybrid founder population used for these experiments. Such clones may have persisted at low frequency under slow growth conditions (D = 0.15 h^-1^) until selection favored them over more abundant, stress-sensitive clones. This possibility highlights an outstanding unresolved issue in experimental evolution, namely the extent to which adaptation results from accumulation of *de novo *mutations as opposed to selection of rare adaptive mutants that may exist at the onset of selection. In this regard, it would be interesting to perform a high-throughput phenotypic screen of hundreds of variants in the founder and terminal populations, as well as to perform population sequencing at the beginning and end of these experiments. Still, given that selection was applied over the course of 500 generations it is virtually certain that multiple *de novo *mutations distinguish members of the terminal populations from their common ancestors.

The industrial applications of hybrid variability have already been recognized [66], and our approach of selecting on a diverse hybrid population may be used to enhance routine industrial strain development. However, our findings highlight the need to carefully choose appropriate parental strains as the selection process cannot rely solely on hybrid vigor. Genome plasticity under strong selection may lead to unexpected results, such as the shedding of one or another parental genome. In our experiments this remarkable occurrence seems to provide the most direct route to thermal tolerance, a trait whose many genetic determinants are widely distributed across the *S. uvarum *and *S. cerevisiae *genomes. Selection on a genetically diverse population of *S. cerevisiae *alone might produce comparable gains in fitness at high temperatures. Indeed, wild isolates of that species have been isolated which can grow at temperatures exceeding 45°C [36], and genome shuffling experiments [67] involving recursive protoplast fusions have produced *S. cerevisiae *strains that aggressively ferment at temperatures up to 48°C [68]. Most importantly, our findings highlight the importance of the ecological theatre in determining the outcome of the evolutionary play. Euploid interspecific *S. cerevisiae *x *S. uvarum *hybrids are genetically stable and highly fit as ambient levels of ethanol increase, but poorly fit under rising temperature. Thus, the evolutionary fate of hybrids in nature likely depends as much on their environmental context as on their genetic potential.

## Methods

### Strains and hybrid creation

All yeast strains used in this work are derivatives of the prototrophic diploid strains CEN.PK (*Saccharomyces cerevisiae*) [44] and CBS7001 (*S. uvarum*) [30] the former obtained from D. Botstein, the latter from E. Louis. To obtain F1 hybrids, *S. cerevisiae *CEN.PK and *S. uvarum *CBS7001 were transformed to G418 and hygromycin antibiotic resistance, respectively, using 2 μ-based YEp352-KanMX and YEp352-hph plasmids. After verifying plasmid segregation, transformants were sporulated for three days on sporulation medium (1% potassium acetate, 0.1% yeast extract and 0.05% glucose) and then mixed and plated on rich medium supplied with G418 and hygromycin at 200 μg mL^-1 ^and 300 μg mL^-1^, respectively. F1 progeny were selected as clones resistant to both antibiotics. After confirming segregational loss of both plasmids, a single F1 clone was sporulated for 3 days in liquid sporulation medium (1% potassium acetate); unsporulated cells were then digested by a combination of Zymolyase T100 and a detergent, as described in [33], leaving F2 hybrid spores, which represent rare viable spore progeny of the F1 clone. These spores were left to germinate and mate overnight, then after verifying cell titer, spread on 48 large plates so that every cell could grow into a colony, unencumbered by others. Approximately 10,000 colonies were washed with 5 mL of sterile ddH_2_O per plate and combined to make the initial hybrid pool.

### Media and growth conditions

Unless otherwise indicated, all media used was the inorganic nitrogen-limiting (0.15 g L^-1 ^(NH_4_)_2_SO_4_) medium used for batch and chemostat cultures described by Verduyn et al. [69]. For chemostat experiments 10 L of basal medium were prepared in 13 L glass carboys. To each liter a post-sterile addition was made of: 1.0 mL 1000× vitamins, 1.0 mL 1000× trace metals, and 45.0 mL 20% glucose (final conc. 9 g L^-1^) [69], a formulation hereafter referred to as low-nitrogen, minimal medium. Populations were cultured in water-jacketed chemostats (200 mL working volume), which were mixed and aerated using sterile, humidified house air at a flow rate of 10 L h^-1 ^(0.8 vvm). For temperature selections, triplicate experimental populations were founded by adding cells from an inoculum prepared as described to a final density of ~10^8 ^cells per mL, with the initial target dilution rate set at D = 0.15 h^-1 ^and the initial temperature at 31°C. Every 25 generations (about every week), culture temperature was increased by 1°C; as cell yield declined steeply above 37°C, later adjustments were made on a bi-weekly basis. To avoid wash-out, dilution rate at higher temperatures was lowered to D = 0.05 h^-1 ^at 41°C and remained at this level until 500 generations. The final vessel temperature at 500 generations was 46.5°C. For ethanol selections, the same founding population was used to inoculate three identical 200 mL chemostat vessels, which were kept at room temperature (25-28°C). The basal medium was identical to that used for temperature selection; ethanol content of this medium was increased by 1% approximately every 10 d. Evaporation was impeded by layering sterile mineral oil atop ethanol-amended, low-nitrogen minimal medium. Because cell growth was strongly inhibited at ethanol amendments > 12%, at these concentrations chemostat dilution rate was reduced to D = 0.05 h^-1 ^to prevent wash-out.

### Sampling and assay of growth parameters

Optical density at λ = 600 nm was measured daily using a Biomate3 spectrophotometer (Thermo Electron Corp, Waltham, MA, USA.) by sterile removal of 1 mL of culture from each vessel, and measuring absorbance of 1:10 dilutions. Approximately every 15 generations 5 mL were removed from each vessel (i) to archive samples as 15% glycerol stocks at -80°C, (ii) to estimate viable cell counts by plating serial dilutions on YPD, and (iii) to determine concentrations of glucose and ethanol, as described below. Three-mL aliquots archived for analysis of residual growth substrate and ethanol were filtered using a 0.2 μm nylon filter and stored at -20°C until assayed.

### Temperature and ethanol tolerance assays

#### Isolates tested

For follow-up experiments we used the diploid parental strains *S. cerevisiae *CEN.PK and *S*. *uvarum *CBS7001, the F1 hybrid, three isolates from the founder hybrid population, and isolates from each of six experimental populations at the final 500-generation time-point. An overview of how the hybrid population was generated and the naming scheme for the isolates is shown in Figure 1, and a detailed list of all tested evolved isolates is presented in Additional file 1: Table S1. Representatives of the founder hybrid population are the same isolates shown in Figure 6A, lanes 1, 2, 3, and are referred to as H1, H2, and H3. Temperature selected isolates tested are shown in Figure 6B Lanes 1, 2, 3 (Temperature selected A1, A2, A3); Lanes 8, 9, 10 (Temperature selected B1, B2, B3); and Lanes 15, 16, 17 (Temperature selected C1, C2, C3). Ethanol evolved isolates tested are shown in Figure 6C Lanes 1, 2, 3 (Ethanol selected D1, D2, D3); Lanes 9, 10, 11 (Ethanol selected E1, E2, E3); and Lanes 16, 17, 18 (Ethanol selected F1, F2, F3). In certain instances, to test the performance of the ancestral haploid form we included a *S. cerevisiae *CEN.PK haploid. For each experimental parameter tested, individual isolates were run in triplicate.

### Growth in liquid media

For all experiments we used parental isolates, F1, 3 founding hybrid isolates, and three isolates from each terminal population (Figure 1). Pre-cultures were grown overnight in 200 Lof low-nitrogen, minimal medium at 25°C and used to inoculate test cultures to a starting A_600 _of 0.01. All test cultures were similarly grown in 200 μL of low-nitrogen, minimal medium in 96-well microtiter plates. To assay temperature tolerance, cultures were grown at 40°C; to test ethanol tolerance cultures were grown at 25°C in the same medium, amended with 8% ethanol. All cultures were grown in triplicate to stationary phase (48 h). Optical density was measured spectrophotometrically at λ = 600 nm every 6 h to confirm that all cultures were in stationary phase before the final measure (Spectramax 340PC, Molecular Devices, Sunnyvale, CA, USA). Replicate estimates of growth parameters for temperature or ethanol isolates (A1-F3) were pooled by vessel for statistical comparison to the parent, F1 and founding strains.

### Induced *versus *inherent thermotolerance

Each parental stain, the F1, the three representative founder stains and two isolates from each temperature selection (isolates 1 and 2 from each vessel in Additional file 1: Table S1) were grown in triplicate overnight at 25°C in 50 mL low-nitrogen, minimal medium to A_600 _0.4-0.6. To test strain-specific differences in induced *versus *inherent thermotolerance, each culture was apportioned into two vessels: one was placed in a 37°C water bath for 5 min and then incubated at 37°C on a shaker (induced thermotolerance), while the other was shaken at room temperature (inherent thermotolerance). After 50 min, cultures were diluted into fresh pre-warmed media, then placed in a 48°C water bath, whereafter samples were removed every hour for 5 h and diluted before plating onto YPD agar. Survivorship was reported as the percentage of viable cells remaining at each time-point, relative to viable cell counts at T = 0 hours.

### Analysis of cell wall phenotypes

To determine whether observed changes in thermal tolerance were correlated with changes in cell wall composition we performed a spheroplast assay, as described in [53]. Cultures were diluted to an A_600 _= 0.8 in spheroplast medium (1.2 M sorbitol), whereupon zymolyase was added to achieve a final assay concentration of 250 μg mL^-1^. Decrease in A_600_, resulting from cell wall digestion, was measured over the course of 1 h at 37°C. All strains were tested in triplicate, and the selected isolates used were the "1" isolates (e.g., A1, B1, etc.) from each evolved population (Additional file 1: Table S1). Strain-specific resistance to Micafungin (Astellas Pharma, Tokyo), a compound that targets fungal cell wall biosynthesis was tested by culturing cells at 25°C in 200 μL of low-nitrogen, with 150 nM Micafungin or a solvent control (DMSO). Cells were inoculated to an A_600 _= 0.01 and incubated until all cultures were in stationary phase and measured spectrophotometrically. Growth was calculated relative to the solvent-only control. All isolates were run in triplicate.

### Genomic analyses

CHEF analysis was conducted as previously described [70,71]. To assay ploidy flow cytometry was performed using SYTOX green as described in [72]. Microarray-based Comparative Genome Hybridization (array-CGH) was performed as described in [30]. Microarray data have been deposited in the GEO repository under accession GSE24479.

#### Statistics

We used one-way ANOVA to compare differences in response to temperature and ethanol tolerance, zymolyase, and Micafungin resistance, using Tukey's HSD. For individual comparisons we used a T-test. We used Sigma Plot 11 (Dundas software LTD, Germany) for all statistical analyses.

## Authors' contributions

JP, EK, SN, AK, and RFR designed and conducted all selection and physiological experiments. EK created the hybrid strains. KC, AS and EK conducted CHEF analysis. BD and GS designed and performed a-CGH analyses. All authors read and approved the final manuscript.

## Supplementary Material

Additional file 1 **Table S1 Founding hybrid and selected isolates used in genetic and physiological experiments**. Reference to each strain's CHEF karyotype is presented is column 2. Click here for file

Additional file 2 **Figure S1 Growth of evolved isolates with 8% ethanol supplementation**. Culture density (A600) of parental, F1, founding and selected hybrid strains from each experimental population following 48 h growth in liquid, low-nitrogen, minimal medium at 25°C in medium, amended with 8% ethanol. Click here for file

Additional file 3 **Figure S2 Assay of Micafungin sensitivity of parental species, founding hybrids, temperature-selected hybrids, and ethanol selected hybrids**. Growth of parental and temperature-selected hybrids in low-nitrogen, minimal medium at 25°C, supplemented with 150 nM of Micafungin. Presented is the growth (A_600_) relative to the solvent (DMSO) control of the 3 replicate cultures in stationary phase. Asterisks indicate that isolates in vessels A and C had significantly greater Micafungin resistance than all other isolates (*P *< 0.05, Mean ± S.E.). Click here for file

Additional file 4 **Figure S3 Changes in ploidy within experimentally selected populations**. Cell populations were stained with SYTOX Green and sorted by flow cytometry as described. **(A) ***S. cerevisiae *haploid and *S. cerevisiae*/*S. uvarum *diploid. **(B) **Vessel A: 101gen (2 N), 157gen (mixed 2 N + 1 N), 224gen (1 N); **(C) **Vessel B: 115gen (2 N), 125gen (mixed 2 N + 1 N), 135gen (1 N); and **(D) **Vessel C: 43gen (2 N), 51gen (mixed 2 N + 1 N), 95gen (1 N). Click here for file

Additional file 5 **Figure S4 CHEF karyotypes in three experimental populations after 400 generations of nitrogen-limited, glucose-sufficient culture with increasing ethanol**. At left are the karyotypes of parental strains, *S. cerevisiae *CEN.PK, *S. uvarum *CBS7001, and their F1 interspecific hybrid. 7 random clones were isolated from each experimental population. Green arrows indicate karyotypic variability in experimental populations. Medium ethanol content at 400 generations was 14%. Click here for file
