# Supplementary material for: Different selective pressures lead to different genomic outcomes as newly-formed hybrid yeasts evolve
Source: BMC Evol Biol. 2012 Apr 2;12:46. doi: 10.1186/1471-2148-12-46 (PMC3372441; doi:10.1186/1471-2148-12-46)
Supplement: Additional file 1 — Table S1 Founding hybrid and selected isolates used in genetic and physiological experiments. Reference to each strain's CHEF karyotype is presented is column 2. [file 1471-2148-12-46-S1.DOCX]

| **Strain** | **Karyotype** |
| --- | --- |
| *S. cerevisiae* CEN.PK | Fig 6a |
| *S. uvarum* CBS7001 | Fig 6a |
| F1 (*S. cerevisiae* x *S. uvarum*) | Fig 6a |
| H1 | Fig 6a Lane 1 |
| H2 | Fig 6a Lane 2 |
| H3 | Fig 6a Lane 3 |
| Temp Selected A1 | Fig 6b Lane 1 |
| Temp Selected A2 | Fig 6b Lane 2 |
| Temp Selected A3 | Fig 6b Lane 3 |
| Temp Selected B1 | Fig 6b Lane 8 |
| Temp Selected B2 | Fig 6b Lane 9 |
| Temp Selected B3 | Fig 6b Lane 10 |
| Temp Selected C1 | Fig 6b Lane 15 |
| Temp Selected C2 | Fig 6b Lane 16 |
| Temp Selected C3 | Fig 6b Lane 17 |
| EtOH Selected D1 | Fig 6c Lane 1 |
| EtOH Selected D2 | Fig 6c Lane 2 |
| EtOH Selected D3 | Fig 6c Lane 3 |
| EtOH Selected E1 | Fig 6c Lane 9 |
| EtOH Selected E2 | Fig 6c Lane 10 |
| EtOH Selected E3 | Fig 6c Lane 11 |
| EtOH Selected F1 | Fig 6c Lane 16 |
| EtOH Selected F2 | Fig 6c Lane 17 |
| EtOH Selected F3 | Fig 6c Lane 18 |
| EtOH Selected 400gen V4-2 | Fig S6 Lane 2 |
| EtOH Selected 400gen V5-3 | Fig S6 Lane 10 |
| EtOH Selected 400gen V6-4 | Fig S6 Lane 18 |

**Supplementary Table 1.** Founding hybrid and evolved isolates used in genetic and physiological experiments. Key to each strain’s CHEF karyotype is presented is column 2
