# Supplementary figures and images for: Different selective pressures lead to different genomic outcomes as newly-formed hybrid yeasts evolve
Source: BMC Evol Biol. 2012 Apr 2;12:46. doi: 10.1186/1471-2148-12-46 (PMC3372441; doi:10.1186/1471-2148-12-46)

Figure S1.

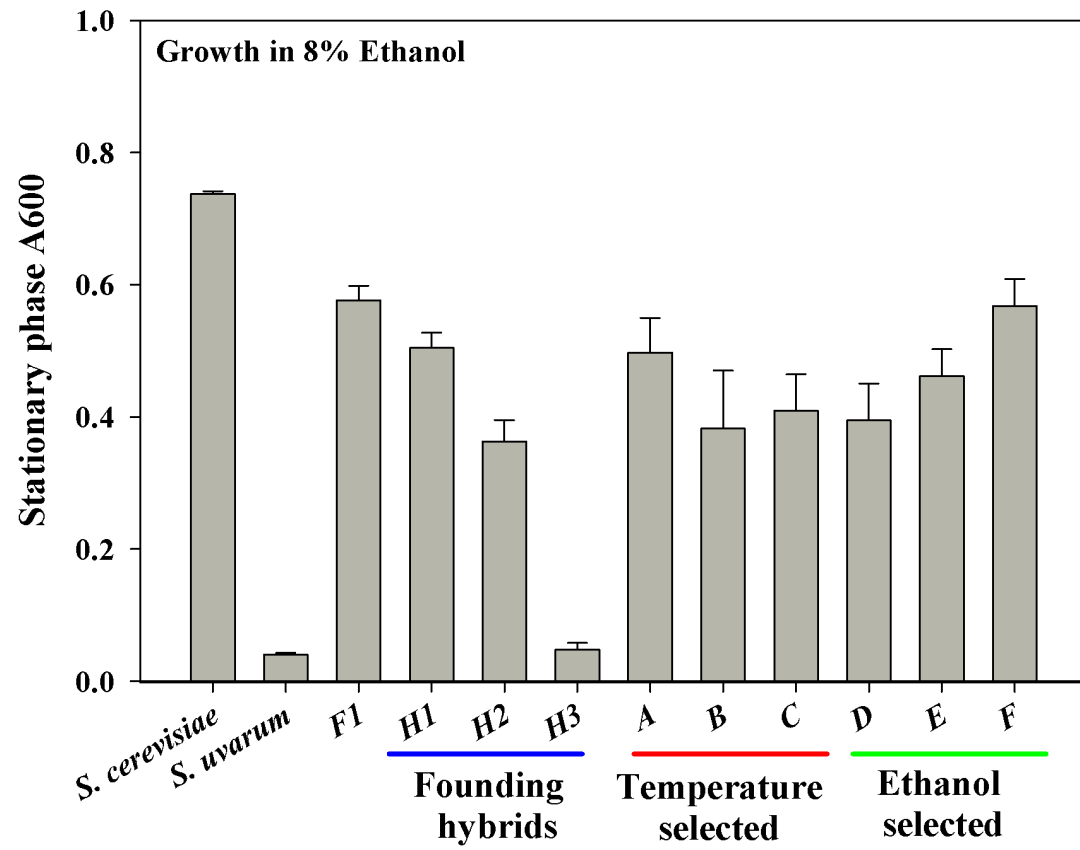

Supplement: Additional file 2 — Figure S1 Growth of evolved isolates with 8% ethanol supplementation. Culture density (A600) of parental, F1, founding and selected hybrid strains from each experimental population following 48 h growth in liquid, low-nitrogen, minimal medium at 25°C in medium, amended with 8% ethanol. [file 1471-2148-12-46-S2.PDF]

**Figure S2.**

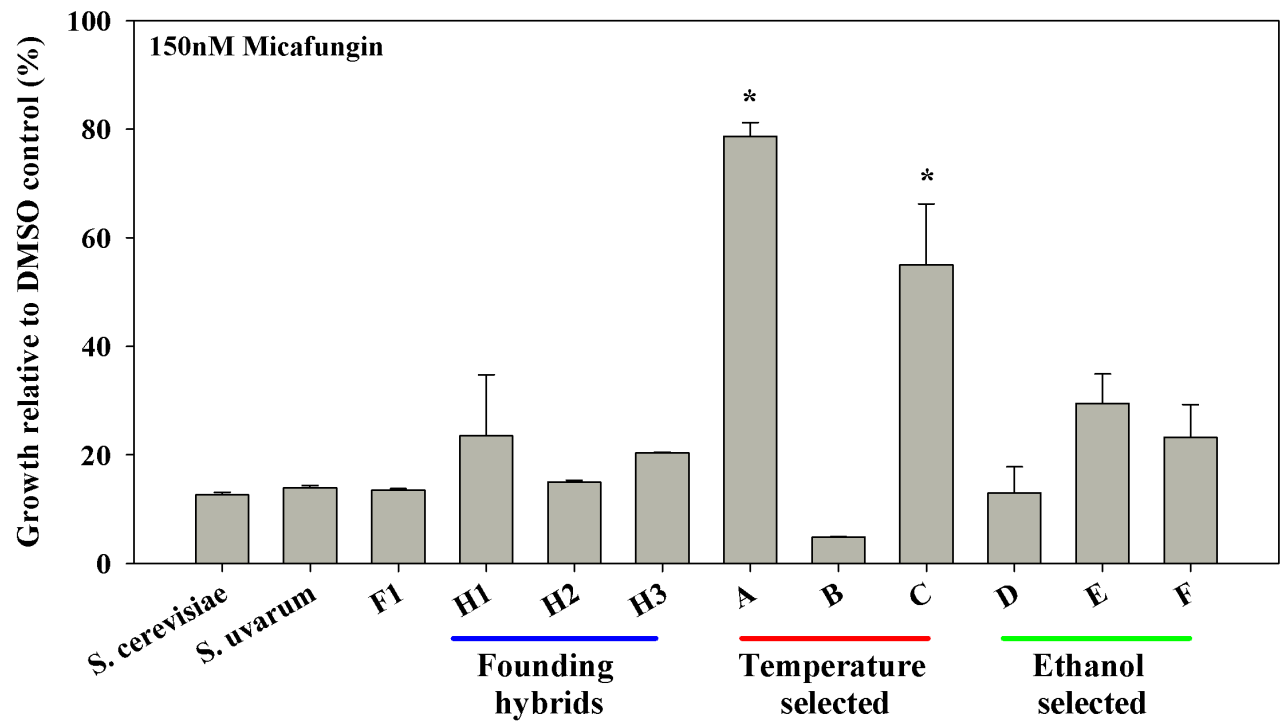

Supplement: Additional file 3 — Figure S2 Assay of Micafungin sensitivity of parental species, founding hybrids, temperature-selected hybrids, and ethanol selected hybrids. Growth of parental and temperature-selected hybrids in low-nitrogen, minimal medium at 25°C, supplemented with 150 nM of Micafungin. Presented is the growth (A600) relative to the solvent (DMSO) control of the 3 replicate cultures in stationary phase. Asterisks indicate that isolates in vessels A and C had significantly greater Micafungin resistance than all other isolates (P < 0.05, Mean ± S.E.). [file 1471-2148-12-46-S3.PDF]

Figure S3.

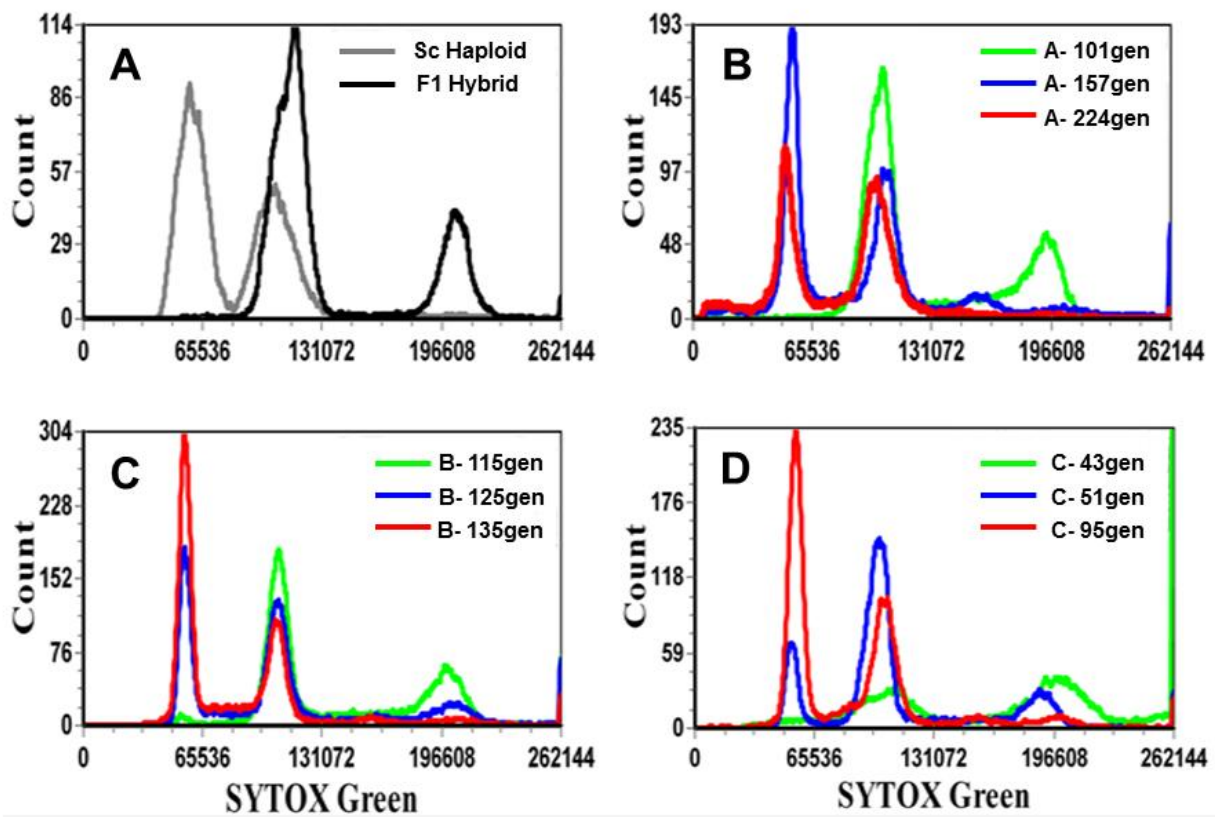

Supplement: Additional file 4 — Figure S3 Changes in ploidy within experimentally selected populations. Cell populations were stained with SYTOX Green and sorted by flow cytometry as described. (A) S. cerevisiae haploid and S. cerevisiae/S. uvarum diploid. (B) Vessel A: 101gen (2 N), 157gen (mixed 2 N + 1 N), 224gen (1 N); (C) Vessel B: 115gen (2 N), 125gen (mixed 2 N + 1 N), 135gen (1 N); and (D) Vessel C: 43gen (2 N), 51gen (mixed 2 N + 1 N), 95gen (1 N). [file 1471-2148-12-46-S4.PDF]

Figure S4.

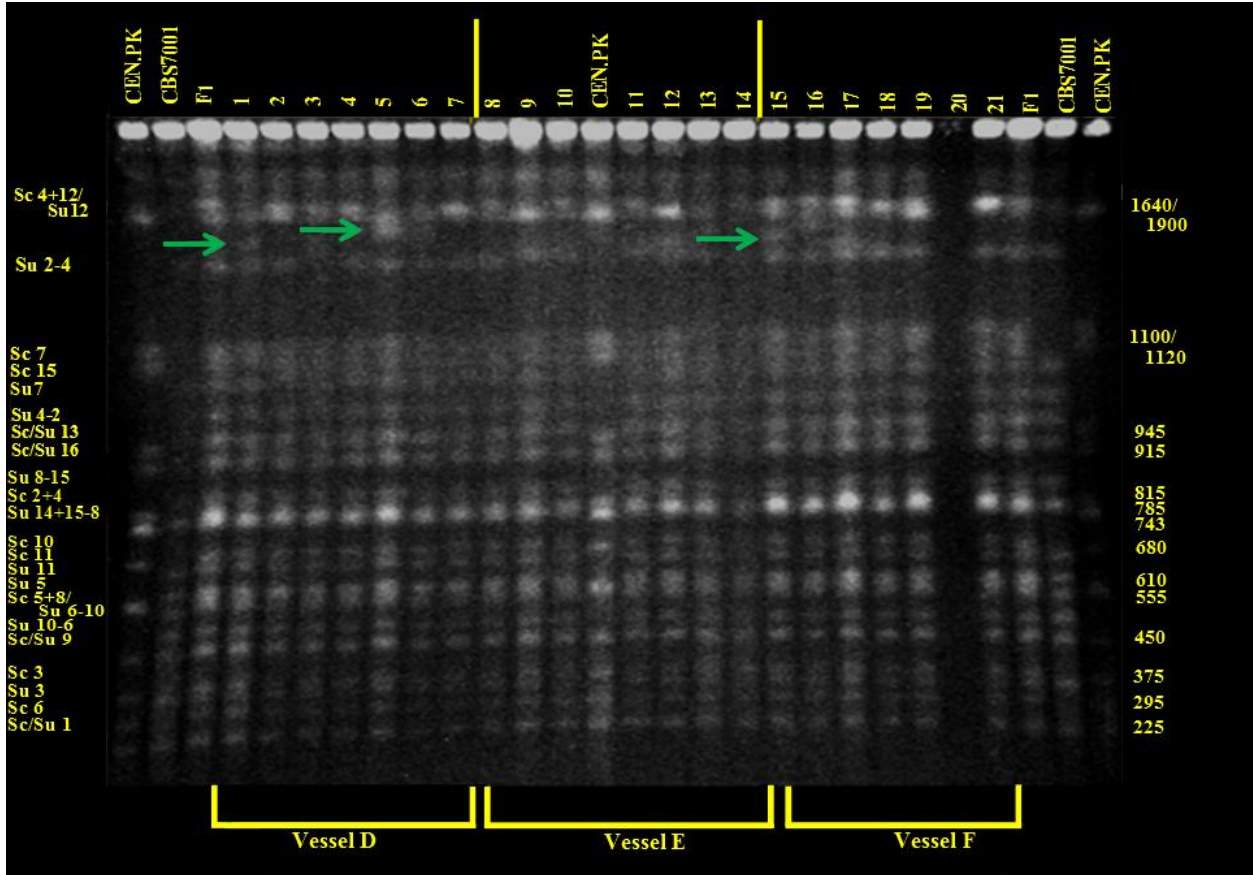

Supplement: Additional file 5 — Figure S4 CHEF karyotypes in three experimental populations after 400 generations of nitrogen-limited, glucose-sufficient culture with increasing ethanol. At left are the karyotypes of parental strains, S. cerevisiae CEN.PK, S. uvarum CBS7001, and their F1 interspecific hybrid. 7 random clones were isolated from each experimental population. Green arrows indicate karyotypic variability in experimental populations. Medium ethanol content at 400 generations was 14%. [file 1471-2148-12-46-S5.PDF]
